# Nationwide analysis of open groin hernia repairs in Italy from 2015 to 2020

**DOI:** 10.1007/s10029-023-02902-z

**Published:** 2023-10-17

**Authors:** M. Ortenzi, E. Botteri, A. Balla, M. Podda, G. Montori, A. Sartori

**Affiliations:** 1 Department of General Surgery, Università Politecnica delle Marche, Piazza Roma 22, 60121 Ancona, Italy; 2 ASST Spedali Civili Di Brescia PO Montichiari, Via Boccalera 325018, Montichiari, Brescia, Italy; 3 Coloproctology and Inflammatory Bowel Disease Surgery Unit, IRCCS San Raffaele Scientific Institute, Via Olgettina 60, 20132 Milan, Italy; 4 Department of Surgical Science, University of Cagliari, Cagliari, Italy; 5 Department of General Surgery, Ospedale Di Vittorio Veneto-ULSS2 Marca Trevigiana, Via Forlanini, 71, 31029 Vittorio Veneto, Treviso Italy; 6 Department of General Surgery, Ospedale Di Montebelluna, Via Palmiro Togliatti, 16, 31044 Montebelluna, Treviso Italy

**Keywords:** Laparoscopic hernia repair, TEP, TAPP, Incarcerated hernias

## Abstract

**Introduction:**

Inguinal hernia repair is one of the most commonly performed operations in general surgery. A total of 130.000 inguinal hernia repairs are performed yearly in Italy, and approximately 20 million inguinal hernias are treated worldwide annually. This report represents the trend analysis in inguinal hernia repair in Italy from a nationwide dataset for the 6-year period from 2015 to 2020.

**Materials and methods:**

Based on regional hospital discharge records, all the inguinal hernia repairs performed in public and private hospitals in Italy between 2015 and 2020 were reviewed based on diagnosis and procedure codes. For the aim of this study, data from the AgeNas (The National Agency for Regional Health Services) data source were analyzed.

**Results:**

Elective inguinal hernia repairs outnumbered urgent operations over the 6-year study period, ranging from 122,737 operations in 2015 to 65,780 in 2020 as absolute numbers, and from 87.96 to 83.3% of total procedures in 2019 and 2020 respectively, with an annual change ranging from − 66.58%, between 2020 and 2019, to − 2.49%, between 2019 and 2018 (mean = − 18.74%; CI =− 46.7%–9.22%; p < 0.0001).

**Conclusions:**

This large-scale review of groin hernia data from a nationwide Italian dataset provides a unique opportunity to obtain a snapshot of open groin hernia repair activity. More specifically, there is a trend to perform more elective than urgent procedures and there is a steady decrease in the amount of open hernia repairs in favor to laparoscopy.

**Supplementary Information:**

The online version contains supplementary material available at 10.1007/s10029-023-02902-z.

## Introduction

Inguinal hernia repair is one of the most commonly performed operations in general surgery. A total of 130,000 inguinal hernia operations are performed annually in Italy, and approximately 20 million are treated worldwide [1, 2]. Open hernia repair is one of the first surgical procedures to have been described and standardized in the late nineteenth century [3]. Due to the proven benefits in reducing recurrence, tension-free mesh repairs have played a central role in the surgical armamentarium for groin hernia repair over the last four decades. In addition, minimally invasive techniques have been successfully incorporated into inguinal hernia surgery and are being used more commonly to date [4].

The choice of surgical technique for inguinal hernia repair remains controversial. Quality assessments of inguinal hernia repair have previously been conducted using two different methods: either through analyzing dedicated regional/national databases (DD) [5–7] or reviewing administrative databases (AD), such as those extracted by Hospital Discharge records regional Databases (HDD) [8]. Data available in Italy are scarce and taken in the most part from surveys and regional datasets [5–10].

Analyzing the data from the AgeNas (The National Agency for Regional Health Services) data source, the present study aimed to offer the first analysis of open inguinal hernia repairs in Italy covering the 6-year period from 2015 to 2020.

## Materials and methods

### Data extraction

Using the Hospital Discharge records regional Databases (HDD), all inguinal hernia repairs carried out in public and private hospitals in Italy in patients over 18 years between 2015 and 2020 were retrieved based on diagnosis and procedure codes. The HDD collected clinical and administrative informations regarding all hospital admissions for patients discharged from any hospital in Italy. The AgeNaS led the management and analysis of data. All hospital admissions for inguinal hernia repair that occurred between 2015 and 2020 were analyzed. The data source included patient demographic data (sex, age) and admission and discharge data with up to six discharge diagnoses [International Classification of Disease, 9th Revision, Clinical Modification (ICD-9-CM)], as well as living status at discharge (alive, dead, transferred to another hospital). It did not provide information on size of the hernia defect, individual surgical expertise, number of surgeons or number of procedures performed per surgeon. It gave information on the use of a mesh for inguinal hernia repair and allowed registration of all readmissions regardless of the primary site of surgery and type of institution (public or private). It did not provide information on hernias treated as outpatient procedures.

The validity of this data system with respect to the type of surgical procedure had been already established for several surgical procedures [5–8]. This study was conducted and reported according to the STROBE (STrengthening the Reporting of OBservational studies in Epidemiology) guidelines [9].

### General data

Informations on consecutive admissions for inpatient inguinal hernia repair in Italy from 2015 to 2020 for patients with a diagnosis of unilateral and bilateral inguinal or femoral hernia were retrieved from the database.

Sex, age and preexisting comorbidities were analyzed. Comorbidities were further divided into general comorbidities and neurological comorbidities. The presence of bowel obstruction at the time of the operation was also registered. The search coding system is summarized Table 1.

**Table 1:** Diagnosis and procedures coding system based on ICD-9-CM codes contained as primary interbentions/diagnosis or among the first five secondary intervention/diagnosis used to search for groin hernia data from 2015 to 2020 (source AgeNas)

	ICD-9-CM diagnosis code	ICD-9-CM treatment code
Unilateral inguinal hernia	550.00; 550.01; 550.02; 550.10; 550.11; 550.90; 550.91	53.00; 53.01; 53.02; 53.03; 53.04; 53.05
Bilateral inguinal hernia	550.00; 550;01; 550.02; 550.10; 550.11; 550.90; 550.91	53.10; 53.11; 53.12; 53.13; 53.14; 53.15; 53.16; 53.17
Unilateral femral hernia	551.00; 551.01; 552.00; 552.01; 553.00; 553.01	53.21; 53.29
Bilateral femoral hernia	552.02; 552.03; 553.03	53.31
Bowel obstruction	55.18; 5528; 55.29	
Comorbidities		
General comorbidities	25.00x (diabetis); 427.31 (atrial fibrillation); 585.9x (kidney failure); 491.20 (respiratory failure); 2865x-V5861 (anticoagulant)	
Neurological comorbidities	33.2xx (Parkinson); 29.00xx-29.03x (dementia); 331.0 (Alzheimer)	
Complications	998.11 (bleeding); 998.12 (hematoma); 998.12 (serohematoma); 99.60x-99.5x (infection) ‘AND’ 998.58–99.89x (wound) OR 996.87 (bowel)	
Associated procedures (AND)		
Cholecystectomy		51.23
Adhesiolysis		5451

### Procedural data

The discrimination between laparoscopic or open groin hernia repair was identified as the presence or the absence of the ICD-9-CM code 54.21 (laparoscopy). The association of inguinal hernia repair with other surgical procedures was defined by the wording "AND cholecystectomy" or "AND adhesiolysis". The presence of an association with another type of surgical procedure other than cholecystectomy and adhesiolysis was grouped as "other". The analysis included total hospital stay, readmission rate within 30 days from the operation, early mortality rate (during the index admission or within 30 days from the operation), late mortality rate (> 30-day mortality) and 30-day morbidity.

Data concerning in-hospital and 30-day mortality were obtained linking the hospital discharge records and the regional registry of mortality through unique patient codes. Complication rates were defined using pre-defined codes either as primary diagnosis or among the first five secondary diagnoses (Table 1).

### Statistical analysis

Data were processed using the MedCal statistical package (version 12.5). Qualitative variables were summarized by absolute numbers and percentages, while quantitative variables were described by the median and standard deviation (SD) or range min–max, for normally or non-normally distributed variables respectively.

Statistical analysis was performed using Student’s t test and the Cochran Armitage test for trends, as appropriate. A two-tailed p value < 0.05 was considered statistically significant.

The annual intervention rate (AIR) per 100,000 population was calculated, assessing the changes in the considered period. The sample size was the Italian population, reported by region, according to the average yearly population on the 31^st^ of December for the period from 2015 to 2020, as reported by the Italian National Institute of Statistics (ISTAT) (Supplemental Table S1) [11].

## Results

### Cohort characteristics

**Fig. 1:**
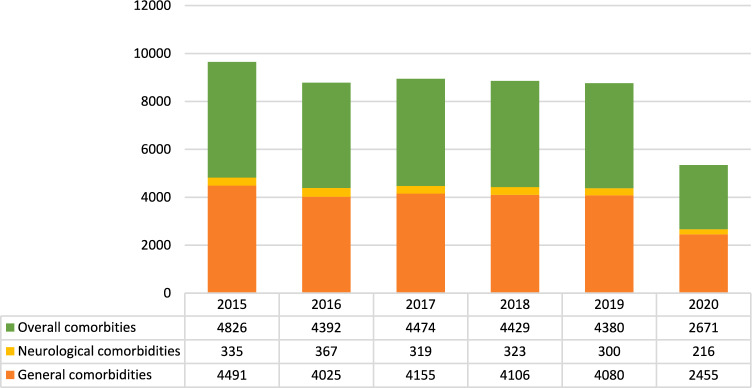
Comorbidities over time in absolute and relative frequencies related to overall comorbidity rate (source AGENAS)

**Fig. 2:**
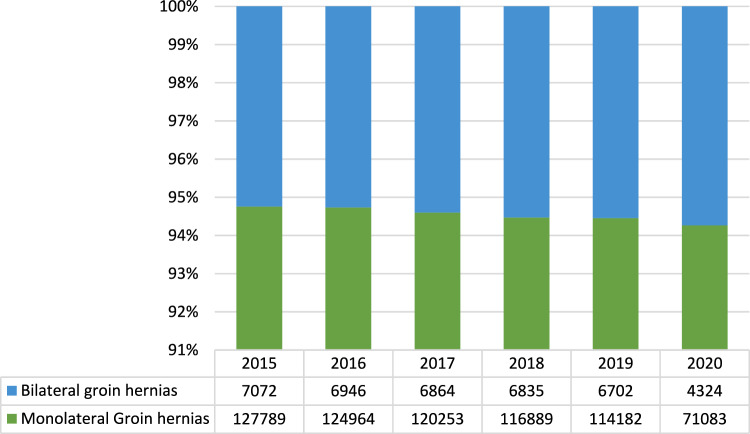
Monolateral and bilateral hernias in absolute and relative frequencies to the whole cohort (source AGENAS)

Male patients represented the majority, although the rate of male patients steadily decreased over time (Range 88.3%—88.8%; Mean annual change − 3.40%; 95%CI − 4.74% to − 2.6%). Mean age ranged from 59 ± 20 years in 2015 to 61 ± 19 years in 2020. Overall comorbidity rates were stable during the study period. More details are shown in Fig. 1. Inguinal hernias were unilateral in 675,160 (93.3%) cases and bilateral in 38,743 (5.3%) cases. Monolateral hernias ranged from a maximum of 122,737 cases in 2015 to a minimum of 65,780 cases in 2020 and from 87.96% to 83.3% in percentage of overall procedures in 2019 and 2020 respectively (Fig. 2**),** with an annual change ranging from a minimum of − 2.26% between 2016 and 2015 to a maximum of – 60.63% between 2019 and 2020 (Mean annual change − 14.4%; 95%CI—37.05% – 8.25; p < 0.0001). The mean annual change was—2.85% (95%CI − 3.59% to—2.11%; p < 0.0001) from 2015 to 2019 (Table 2).

**Table 2:** Annual change observed in the considered period

	2016	2017	2018	2019	2020	Mean	SD	CI 95%
Monolateral	− 2,26	− 3,91	2,87	− 2,37	− 60,63	− 14.41	25.84	22.65
Bilateral	− 1,81	− 1,19	− 0,42	− 1,98	− 54,99	− 12,07	23.99	21.03

Of this cohort, 0.02% patients (n = 171 over time) presented with bowel obstruction.

### Procedural data

**Fig. 3:**
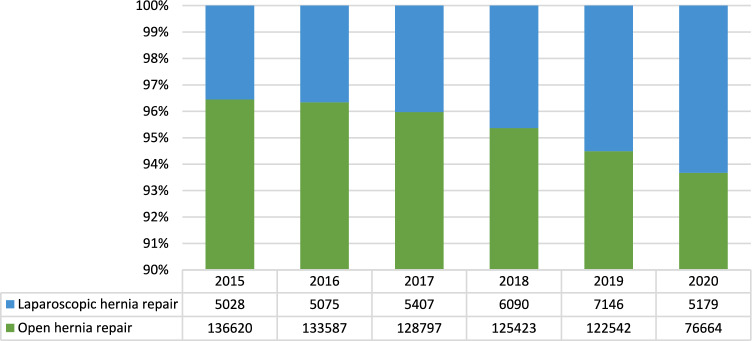
Open and laparoscopic hernia repairs in absolute and relative frequencies performed in the index period (source AGENAS)

A total of 757,558 inpatient inguinal hernia repairs were performed in Italy from 2015 to 2020. Of these, 723,633 (95.5%) were open repairs (Table 3, Fig. 3).

**Table 3:** Annual change observed in the considered period

	2015	2016	2017	2018	2019	Mean	SD	CI 95%
Open hernia repair	− 2.27	− 3.71	− 2.69	− 2.35	− 59.84	− 14.17	25.53	22.38
Laparosocpic hernia repair	0.92	6.14	11.21	14.77	37.98	− 0.98	21.33	18.69

The number of open procedures performed steadily decreased over time with the maximum annual change of − 59.84% observed between 2019 and 2020. The mean annual decrease in the 6-year study period was − 14.17% (95%CI: − 36.55%—8.21%; p < 0.0001), but, excluding 2020, the numbers of open procedures decreased steadily with a mean annual change of − 2.75% (95%CI − 3.39% to—2.11%; p < 0.0001) (Table 4).

**Table 4:** Annual change observed in Elective and Urgent procedures in the considered period

	2016	2017	2018	2019	2020	Mean	SD	CI 95%
Urgent	− 3.03	− 3.80	0.97	− 1.09	− 19.12	− 1,74	2.14	1.87
Elective	− 2.18	− 3.70	− 2.49	− 2.49	− 66.57	− 2,72	0.67	0.59

In contrast, an overall steady increasing trend was observed in the number of procedures performed laparoscopically over time from a minimum of 5,028 (3.55% of all procedures performed in 2015) to a maximum of 7,146 (5.51% of all procedures performed in 2019). The mean annual change was 8.26% (95%CI 2.35%—14.17%) from 2015 to 2019, while a mean annual change of − 0.98% (95%CI: − 19.68%—17.72%) was observed over the whole time period (Fig. 3).

**Fig. 4:**
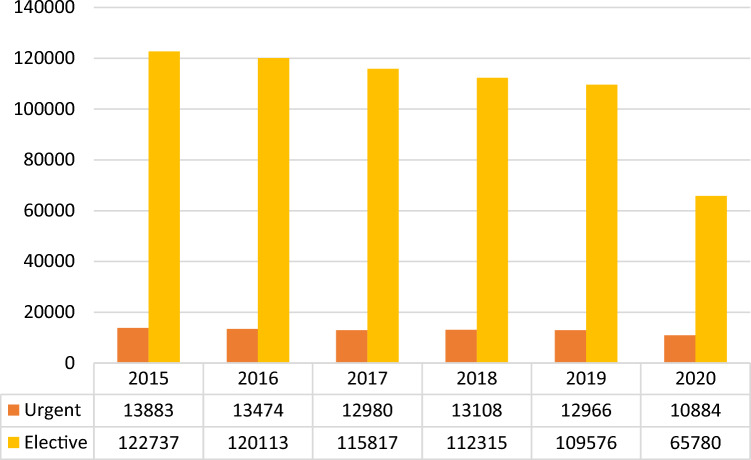
Elective and urgent procedures in absolute frequencies (source AGENAS)

Elective operations outnumbered urgent operations ranging from 122,737 cases in 2015 to 65,780 cases in 2020 and from 87.96% to 83.3% of overall procedures in 2019 and 2020 respectively, with an annual change ranging from a maximum of − 66.58%, between 2020 and 2019, to a minimum of– 2.49%, between 2019 and 2018, (Mean annual change -18.74%; 95%CI − 46.7%–9.22%; p < 0.0001) (Fig. 4) The mean annual decrease was − 2.79% (95%CI − 3.58% to—2%; p < 0.001) from 2015 to 2019 (Table 4).

Urgent procedures ranged from 10,884 cases in 2020 to 13,883 cases in 2015 and from 9.92% to 13.8% of overall procedures in 2017 and 2020 respectively (Mean annual change − 1.73%; CI = − 3.6% – 0.14%). The mean annual decrease was – 1.95% (CI: − 4.45%—0.55%; p < 0.0001) from 2015 to 2019 (Table 4).

A total of 14,335 (1.98%) inguinal hernia repairs were associated with additional surgical procedures between 2015 and 2020. Of these, 2,914 (0.43%) were associated with cholecystectomy, 1,477 (0.20%) with adhesiolysis, and 9,601 (1.42%) with other types of open surgical procedures. The mean annual change of this trend was − 1.23% (95%CI − 4.45%—0.55%) if considering the total number of the associated procedures.

The trend of associated cholecystectomies varied with a mean annual decrease of – 11.32 (95%CI − 28.1%—5.46%; p = 0.165) over time, and of − 3.12% (95%CI − 10.77%—4.53%; p = 0.886) from 2015 to 2019. The association of open inguinal repair and adhesiolysis demonstrated a mean annual change of 3.55% (95%CI − 6.86%—13.96%; p < 0.0001), decreasing to 2.85% (95%CI − 10.47%—16.17%; p < 0.0001) from 2015 to 2019.

### Complications and readmission

**Fig. 5:**
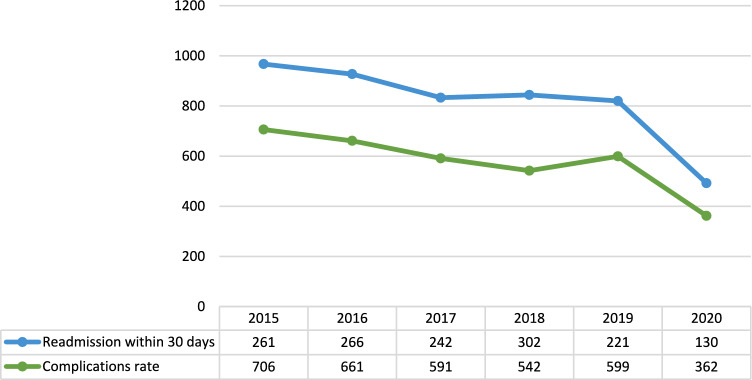
Complications and readmissions within 30 days rates from operation (source AGENAS)

A total of 3461 complications were registered, ranging from 362 in 2020 to 706 in 2015 as absolute numbers and from 0.43% to 0.52% in relative frequencies of overall procedures in 2018 and 2015, respectively. Complication rates decreased over time, with an annual change ranging from − 6.4% between 2016 and 2015 to − 65% between 2020 and 2019 (Mean annual change − 20.34%; 95%CI—42.28% – 1.6%; p = 0.0723). The mean annual change was − 9.18% (95%CI − 11.2% to—7.16%; p = 0.079) from 2015 to 2019 (Fig. 5).

A total of 1422 readmissions were registered, ranging from 130 in 2020 to 302 in 2018 as absolute frequencies and from 0.24% to 0.17% in relative frequencies of overall procedures in 2018 and 2020 respectively, with a mean annual change ranging from − 70% between 2020 and 2019 to 19.86% between 2017 and 2016 (Mean annual change − 15.57%; 95%CI − 49.15% – 18.01%; p = 0.5752). The mean annual change was − 1.96% (95%CI -28.31% – 24.39%; p = 0.568) between 2015 and 2019 (Fig. 5).

### Mortality

A total of 2500 deaths were registered, 1347 (53.9%) within 30 days of the operation and 1153 after 30 days (42.7%), with a mean annual change ranging from − 13.3% between 2016 and 2015, and 0.25% between 2020 and 2019 (Mean annual change − 3.63%; 95%CI—19.09 – 11.83; p < 0.001). The mean annual change was − 4.60% (95%CI − 16.82% – 7.62%; p < 0.0001) from 2015 to 2019.

Early mortality ranged from 194 in 2016 to 254 in 2015 as absolute frequencies and from 0.48% to 0.57% in relative frequencies of overall procedures in 2018 and 2020 respectively, with an annual change ranging from − 30.9% between 2016 and 2015 to 10.29% between 2018 and 2017 (Mean annual change − 3.67%; 95%CI =—19.72% – 12.35%; p < 0.0001). The mean annual decrease was − 5.92% (95%CI − 25.84% – 14%; p = 0.526) from 2015 to 2019.

**Fig. 6:**
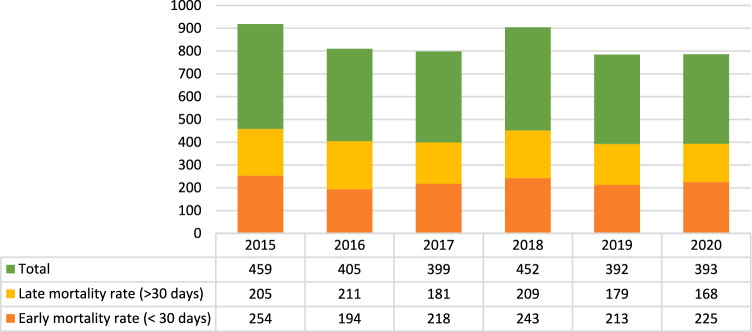
Early and late mortality rates in absolute and relative frequencies related to overall mortality rate (source AGENAS)

Late mortality ranged from 168 in 2020 to 211 in 2016 as absolute frequencies and from 0.14% to 0.21% in relative frequencies of overall procedures in 2017 and 2020 respectively, with a mean annual change ranging from − 16.76% between 2018 and 2017 to 13.39% between 2018 and 2017 (Mean annual change − 4.71%; 95%CI = − 16.06% – 6.65%; p = 0.0108). The mean annual change was − 4.26% (95%CI − 25.21% – 16.69%; p = 0.976) if considering from 2015 to 2019. Figure 6

### Techniques and pathways

**Fig. 7:**
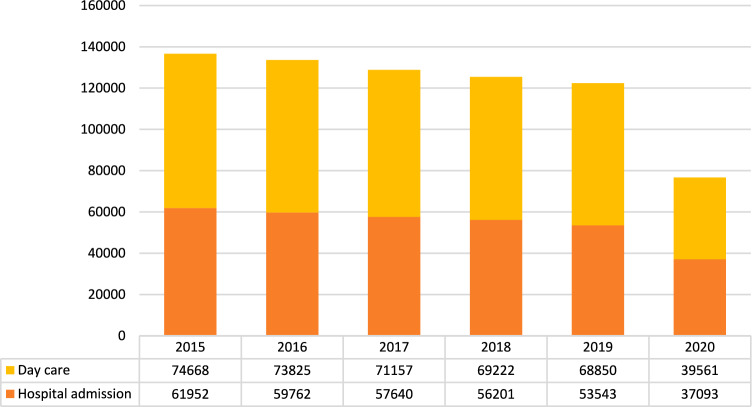
Type of hospital admission in absolute and relative frequencies in the index period (source AGENAS)

Concerning the type of healthcare provided, the number of procedures performed in inpatient admission ranged from 37,093 in 2020 to 61,952 in 2015 and from 43.69% to 48.38% of overall procedures in 2019 and 2020 respectively, with a mean annual change ranging from a maximum of − 44.4% between 2020 and 2019 to a minimum of − 2.56% between 2018 and 2017 (Mean annual change − 11.84%; 95%CI − 29.66 – 5.96; p < 0.0001) (Table 5). The mean annual change was − 3.71% (CI: − 33.18% – 25.76%; p < 0.0001) between 2015 and 2019 (Fig. 7).

**Table 5:** Annual change observed in Elective and Urgent procedures in the considered period

	2016	2017	2018	2019	2020	Mean	SD	CI 95%
Urgent	− 3.03	− 3.80	0.97	− 1.09	− 19.12	− 1,74	2.14	1.87
Elective	− 2.18	− 3.70	− 2.49	− 2.49	− 66.57	− 2,72	0.67	0.59

*SD* standard deviation, *CI* confidence interval

The number of operations managed as day surgery procedures ranged from 39,561 in 2020 to 74,668 in 2015 as absolute frequencies and from 51.6% to 56.18% as relative frequencies of overall procedures in 2020 and 2019 respectively, with an annual change ranging from − 74% between 2020 and 2019 to − 0.54% between 2019 and 2018 (Mean annual change − 16.44%; 95%CI − 47.99 – 15.11). The mean annual change was − 2.05% (95%CI − 4.43% – 0.33%) between 2015 and 2019. Figure 7

Mean hospital stay was 5 days between 2015 and 2019 and 4 in 2020*.*

Regional data are provided as supplemental material.

## Discussion

This study provides a picture of the epidemiological trends over a six-year period for open inguinal hernia repair surgery in Italy. Although there have been previous studies involving ‘‘hernia audits’’ in regional populations [5, 9, 10], nationwide studies have never been conducted. Some of the main aspects of clinical interest include the nature of the initial hospital admission and postoperative recovery time. In many European countries, including England [12], there are strict surgical guidelines for hernia repair.

Currently, however, the treatment of inguinal hernias is not standardized across Europe and the choice of the surgical approach remains debatable and subject to bias, including individual surgeon experience and preference. The open approach remains prevalent in both elective and emergency settings worldwide [13–19], consistent with our database findings.

The results reported in both elective and emergency inguinal hernia repairs performed between 2015 and 2020 are consistent with the literature [9, 20, 21]. Overall hernia numbers have trended towards a gradual, yet significant decline over the years, as seen in other countries [22].

Primarily, there was a gradual and significant decline in the number of open hernia repairs both in elective (p < 0.0001) and emergent setting (p < 0.0001). This decrease coincided, in part, with the increase in use of laparoscopy, and, additionally, with the gradual decline of inpatient hospital stay for groin repair (p < 0.001). Similarly, a reduction in the number of admissions for inpatient surgery was noted (p < 0.0001). [23]

Other international studies have noted a similar reduction in the use of inpatient procedures due to several factors, including broader use of local anesthesia for elective inguinal hernia repair [24], which has coincided with higher rates of day-surgery patients [25] and less restrictive selection criteria for eligibility for day surgery.

The reduction in inpatient directed care, probably coincided with an increase in the ambulatory service. The choice of ambulatory service could be further investigated; however, other authors have reported the steady implementation of ambulatory services into their routine practice [9, 22].

The otherwise well-established ambulatory set-up for elective groin hernia repairs [22, 26–28] has only been gradually implemented nationwide. However, numbers have increased from approximately 55% in 1998 to around 70% in 2005. A large variability in the implementation of an ambulatory set-up has been demonstrated, reflecting the difficulties in changing traditional practice based on available scientific evidence [20, 26–28].

Complications and readmission rates did not significantly change in the considered period, when the decrease observed reflects the overall decrease of the overall procedures. A large study conducted in Denmark identified a hospital readmission rate for hernia of 1.8% [29]. In our cohort the readmission rate was as high as 0.42%.

Our data also demonstrated that the number of days spent in hospital for elective admissions did not change over the decade considered. A likely explanation for this is that, despite gradual improvements in healthcare provision, the greater selection of patients referred for elective hospital admissions over the years has altered the case mix.

In-hospital mortality remained relatively constant. A significant reduction in both early and late mortality rates was observed across the whole period but more significantly decreased in the period between 2015 and 2019. This trend reflected the drastic drop of hernia cases in 2020.

The global necessity to adapt to the unprecedented challenges associated with the rapid spread of Sars-Cov-2 in early 2020 resulted in a significant disruption in all elective and benign surgery. Over a few short weeks, surgeons witnessed a dramatic change in their practice, with a rapid decrease in the number of surgical interventions reported worldwide [30].

General surgeons were particularly affected due to the wide variety of procedures they perform, many of which are carried out routinely, many in outpatient settings [30].

A nationwide survey [30] highlighted that Italy, together with other countries, faced a dramatic drop in surgical activity, with reports of operative activity more than halving or entirely suspended by the 64% and 15% of the surgeons participating in the survey, respectively. Other authors reported similar data focusing on the fact that most of the operative reduction burdened elective procedures, with an overall reduction of over 20% of normal surgical activity [31]. The data of the present study demonstrated a reduction of − 59.84% of overall procedures between 2019 and 2020 mainly explained by the drop of − 66.58% in elective procedures registered in 2020, in contrast a more modest reduction of − 6.24% was observed in urgent procedures. The drastic reduction in hernia surgery cases presents significant implications for surgical recovery planning over the coming years.

The limit of this paper is intrinsic in the search methodology and the data source. The system involves AD that collect data from all public and private hospitals of a given region or country, obtaining such information from hospital admissions and discharge charts. The main advantage of the AD system is the ability to retrospectively record almost 100% of the surgical procedures performed using the coding system based on the ICD-9-CM. Additionally, AD are readily accessible at low cost. However, they often result in inaccurate data registration as well as inexact follow-up and recurrence/complication information; additionally, they often lack case mix adjustment.

Inguinal hernia repair could be used as an indicator of the surgical quality offered in different health institutions and countries, thereby establishing a scientific basis from which to make decisions, given the rate with which day surgery and outpatient surgery are developing. Such solutions could help improve elective surgery efficiency, better sustainability across healthcare services, and offer elective patients shorter waiting times. Finally, this dataset can be used as a tool to help present validated recommendations for the management of patients with groin hernias, with a potential to implement a registry of ambulatory hernia repairs, following the example of other European countries.

## Conclusions

This large-scale review of inguinal hernia data from a nationwide Italian dataset provides a unique opportunity to obtain a snapshot of inpatient open hernia repair activity. Generally, a steady substantial decrease of open procedures could be observed over the six-year period taken into consideration and a simultaneous increase of laparoscopic adoption, in the setting of a global reduction observed in 2020 due to the COVID pandemic. The trend of day care surgeries remained stable throughout the whole period.

### Supplementary Information

Below is the link to the electronic supplementary material.

Supplementary file1 (DOCX 19 KB)

Supplementary file2 (DOCX 25 KB)

Supplementary file3 (DOCX 28 KB)

Supplementary file4 (DOCX 944 KB)

Supplementary file5 (DOCX 630 KB)

Supplementary file6 (DOCX 942 KB)

Supplementary file7 (DOCX 941 KB)

Supplementary file8 (DOCX 19 KB)

## Data Availability

Data are available at AgeNas.
