# Supplementary material for: Nationwide analysis of open groin hernia repairs in Italy from 2015 to 2020
Source: Hernia. 2023 Oct 17;27(6):1429–37. doi: 10.1007/s10029-023-02902-z (PMC10700422; doi:10.1007/s10029-023-02902-z)
Supplement: Supplementary file 2 — Supplementary file2 (DOCX 25 KB) [file 10029_2023_2902_MOESM2_ESM.docx]

*Supplemental Table 2 Annual Intervention rate (AIR) for elective procedures per 100,000 inhabitants (Region A; Macroregion B; C annual change) The number of elective procedures steadily decreased across Italy (p<0.001) with the minimum mean decrease of -8.1% (CI: − 16.05 % - -1.05 %; p <0.001) registered in the Abruzzo region and the maximum mean decrease of -32.97% (CI: − 75.62 % - 9.68 %; p <0.001) observed in Valle d’Aosta, when considering the whole time period. However, from 2015 to 2019, the minimum mean decrease of -0.22% (CI: − 2.26 % to – 1.82%; p= ) was observed in the Lombardy region and the maximum mean decrease of -11.32% (CI: −16.93% - - 5.71%; p <0.001) was observed in Valle d’Aosta*.

| **Region** | **Year** | | | | | |
| --- | --- | --- | --- | --- | --- | --- |
|  | **2015** | **2016** | **2017** | **2018** | **2019** | **2020** |
| **Piemonte** | 276 | 274 | 258 | 249 | 245 | 128 |
| **Valle d'Aosta** | 243 | 210 | 181 | 169 | 162 | 74 |
| **Lombardia** | 272 | 269 | 269 | 262 | 268 | 147 |
| **Trentino Alto Adige** | 209 | 203 | 188 | 166 | 172 | 123 |
| **Veneto** | 99 | 99 | 84 | 85 | 86 | 64 |
| **Friuli Venezia Giulia** | 272 | 257 | 261 | 261 | 248 | 176 |
| **Liguria** | 175 | 168 | 147 | 140 | 151 | 79 |
| **Emilia-Romagna** | 243 | 239 | 241 | 248 | 239 | 153 |
| **Toscana** | 294 | 289 | 289 | 294 | 294 | 185 |
| **Umbria** | 315 | 293 | 274 | 260 | 256 | 169 |
| **Marche** | 270 | 259 | 261 | 244 | 250 | 159 |
| **Lazio** | 229 | 215 | 200 | 196 | 195 | 140 |
| **Abruzzo** | 270 | 279 | 251 | 244 | 229 | 191 |
| **Molise** | 229 | 200 | 206 | 207 | 218 | 151 |
| **Campania** | 237 | 228 | 226 | 194 | 163 | 92 |
| **Puglia** | 161 | 154 | 141 | 146 | 140 | 91 |
| **Basilicata** | 271 | 258 | 249 | 243 | 211 | 126 |
| **Calabria** | 122 | 118 | 114 | 121 | 114 | 76 |
| **Sicilia** | 141 | 151 | 138 | 127 | 137 | 98 |
| **Sardegna** | 303 | 299 | 296 | 321 | 295 | 218 |

**A**

| Macroregion | Year | | | | | |
| --- | --- | --- | --- | --- | --- | --- |
|  | **2015** | **2016** | **2017** | **2018** | **2019** | **2020** |
| Northern Italy | 226 | 223 | 216 | 213 | 213 | 125 |
| Central Italy | 291 | 249 | 241 | 238 | 237 | 159 |
| Southern italy | 96 | 92 | 89 | 83 | 74 | 44 |
| Island | 181 | 187 | 177 | 175 | 176 | 127 |

**B**

|  | 2015 | 2016 | 2017 | 2018 | 2019 | MEAN | SD | CI 95% |
| --- | --- | --- | --- | --- | --- | --- | --- | --- |
| Piemonte | -1,119 | -6,624 | -4,193 | -2,258 | -93,218 | -21,48 | 40,16 | 35,20 |
| Valle d'Aosta | -16,165 | -16,157 | -8,019 | -4,950 | -119,565 | -32,97 | 48,66 | 42,65 |
| Lombardia | -0,749 | 0,175 | -2,664 | 2,341 | -82,529 | -16,69 | 36,85 | 32,30 |
| PA di Bolzano | -2,004 | -12,057 | -8,462 | -11,908 | -53,524 | -17,59 | 20,50 | 17,97 |
| PA di Trento | -3,560 | -3,691 | 1,688 | -1,979 | -32,952 | -8,10 | 14,06 | 12,33 |
| Veneto | -0,580 | -18,115 | 1,566 | 1,120 | -33,662 | -9,93 | 15,59 | 13,66 |
| Friuli Venezia Giulia | -6,328 | 1,550 | 0,063 | -5,643 | -41,541 | -10,38 | 17,76 | 15,56 |
| Liguria | -4,516 | -15,313 | -5,248 | 6,391 | -90,555 | -21,85 | 39,17 | 34,33 |
| Emilia-Romagna | -1,887 | 1,175 | 3,213 | -3,677 | -57,617 | -11,76 | 25,77 | 22,59 |
| Toscana | -1,983 | -0,187 | 1,516 | -0,350 | -59,170 | -12,03 | 26,38 | 23,12 |
| Umbria | -8,107 | -7,283 | -5,952 | -1,887 | -52,257 | -15,10 | 20,91 | 18,33 |
| Marche | -4,790 | 0,527 | -7,291 | 1,589 | -58,232 | -13,64 | 25,20 | 22,09 |
| Lazio | -6,006 | -7,588 | -1,790 | -1,295 | -39,358 | -11,21 | 15,97 | 13,99 |
| Abruzzo | 2,861 | -11,993 | -3,441 | -6,775 | -21,151 | -8,10 | 9,07 | 7,95 |
| Molise | -15,097 | 2,377 | -0,159 | 3,817 | -47,191 | -11,25 | 21,46 | 18,81 |
| Campania | -4,046 | -1,059 | -17,283 | -19,362 | -80,710 | -24,49 | 32,43 | 28,42 |
| Puglia | -4,821 | -9,576 | 2,515 | -4,631 | -55,827 | -14,47 | 23,52 | 20,62 |
| Basilicata | -5,681 | -4,208 | -3,469 | -15,911 | -69,913 | -19,84 | 28,44 | 24,93 |
| Calabria | -3,592 | -4,342 | 5,608 | -7,414 | -52,294 | -12,41 | 22,82 | 20,00 |
| Sicilia | 6,082 | -9,927 | -9,823 | 6,743 | -41,170 | -9,62 | 19,43 | 17,03 |
| Sardegna | -1,633 | -1,282 | 7,036 | -9,424 | -37,439 | -8,55 | 17,17 | 15,05 |

**C**
