# Supplementary material for: Nationwide analysis of open groin hernia repairs in Italy from 2015 to 2020
Source: Hernia. 2023 Oct 17;27(6):1429–37. doi: 10.1007/s10029-023-02902-z (PMC10700422; doi:10.1007/s10029-023-02902-z)
Supplement: Supplementary file 3 — Supplementary file3 (DOCX 28 KB) [file 10029_2023_2902_MOESM3_ESM.docx]

Supplemental Table 3 Annual Intervention rate (AIR) for urgent procedures per 100,000 inhabitants (Region A; Macroregion B, C annula trend) Considering urgent procedures, a significative decrease (p<0.001) was also observed across Italy over the considered period with a minimum mean increase of -2.39% (CI: − 30.74% - 26.02%; p <0.001) registered in the Abruzzo region and a maximum mean decrease of -21.62% (CI: − 54.29 % - 11.05 %; p <0.001) observed in Valle d’Aosta, over the whole considered period. However, from 2015 to 2019, the number of urgent open groin hernia repairs increased in 5 regions with a maximum mean increase of 5.79% (CI: −16.93% - - 5.71%; p <0.001) observed in Abruzzo, and a maximum mean decrease of -9.84% (CI: 39.66% to – 19.98%; p= ) in Valle d’Aosta. More generally, the maximum mean decrease was observed in Southern Italy both when considering the whole period and between 2015 to 2019 only (-17.29%; CI: −37.94 % - 3.36 %; p <0.001) and -6.86; - CI: −10.47% - -3.25 %; p<0.001). Regarding elective procedures; the maximum mean decrease was observed in Sicily and Sardinia over the whole time period (-64.67%; CI: −190.87 % - 61.53 %; p <0.001) and in Central Italy (-2.59%; CI: −16.7 % - 11.52%; p<0.0001) between 2015 and 2019.

|  | Year | | | | | |
| --- | --- | --- | --- | --- | --- | --- |
| Region | **2015** | **2016** | **2017** | **2018** | **2019** | **2020** |
| Piemonte | 20 | 20 | 20 | 18 | 20 | 15 |
| Valle d'aosta | 28 | 29 | 25 | 17 | 22 | 13 |
| Lombardia | 18 | 17 | 16 | 15 | 16 | 13 |
| Trentino alto adige | 18 | 17 | 18 | 15 | 15 | 14 |
| Veneto | 15 | 16 | 14 | 15 | 15 | 13 |
| Friuli venezia giulia | 13 | 14 | 13 | 13 | 12 | 10 |
| Liguria | 24 | 23 | 23 | 23 | 20 | 16 |
| Emilia-romagna | 19 | 18 | 18 | 18 | 16 | 13 |
| Toscana | 26 | 26 | 25 | 23 | 22 | 18 |
| Umbria | 39 | 37 | 41 | 38 | 38 | 30 |
| Marche | 21 | 20 | 22 | 21 | 21 | 17 |
| Lazio | 25 | 23 | 15 | 23 | 20 | 19 |
| Abruzzo | 27 | 31 | 24 | 42 | 42 | 31 |
| Molise | 48 | 52 | 52 | 56 | 45 | 37 |
| Campania | 28 | 27 | 30 | 27 | 27 | 23 |
| Puglia | 32 | 32 | 33 | 33 | 32 | 28 |
| Basilicata | 44 | 47 | 47 | 50 | 52 | 40 |
| Calabria | 30 | 22 | 25 | 24 | 23 | 20 |
| Sicilia | 23 | 24 | 23 | 20 | 24 | 23 |
| Sardegna | 23 | 26 | 26 | 24 | 23 | 23 |

**A**

|  | Year | | | | | |
| --- | --- | --- | --- | --- | --- | --- |
| MACRoRegion | **2015** | **2016** | **2017** | **2018** | **2019** | **2020** |
| Northern Italy | 18 | 18 | 17 | 17 | 16 | 13 |
| Central Italy | 26 | 25 | 21 | 25 | 24 | 20 |
| Southern Italy | 31 | 29 | 31 | 30 | 29 | 25 |
| IslandS | 23 | 24 | 24 | 21 | 24 | 6 |

**B**

|  | 2015 | 2016 | 2017 | 2018 | 2019 | Mean | SD | CI 95% |
| --- | --- | --- | --- | --- | --- | --- | --- | --- |
| Piemonte | 1,352 | -2,900 | -8,427 | 5,469 | -35,426 | -7,986 | 16,181 | 14,183 |
| Valle d'Aosta | 5,405 | -19,354 | -47,619 | 22,222 | -68,75 | -21,619 | 37,265 | 32,663 |
| Lombardia | -4,423 | -6,289 | -2,207 | 1,534 | -23,930 | -7,063 | 9,868 | 8,650 |
| PA di Bolzano | 14,285 | 14,953 | -24,418 | 0 | -8,860 | -6,522 | 14,914 | 13,072 |
| PA di Trento | 14,736 | -11,764 | -14,864 | 1,333 | -10,294 | -4,171 | 12,220 | 10,711 |
| Veneto | 2,906 | -10,029 | 6,394 | -1,800 | -11,076 | -2,721 | 7,727 | 6,773 |
| Friuli Venezia Giulia | 10,919 | -9,433 | 0 | -12,76 | -21,551 | -6,566 | 12,450 | 10,913 |
| Liguria | -1,657 | -1,117 | 0,555 | 20,805 | -22,131 | -9,031 | 11,392 | 9,985 |
| Emilia-Romagna | -4,119 | -2,429 | 2,005 | -8,571 | -22,704 | -7,164 | 9,476 | 8,306 |
| Toscana | -0,841 | -1,385 | -10,613 | -5,603 | -23,348 | -8,359 | 9,253 | 8,110 |
| Umbria | -7,763 | 11,294 | -10 | 1,492 | -29,343 | -6,864 | 15,129 | 13,260 |
| Marche | -2,597 | 6,666 | -4,430 | 1,25 | -27,490 | -5,320 | 13,101 | 11,483 |
| Lazio | -9,403 | -54,609 | 35,222 | 10,771 | -10,808 | -10,074 | 31,767 | 27,844 |
| Abruzzo | 12,098 | -31,493 | 42,962 | -0,371 | -35,175 | -2,396 | 32,375 | 28,378 |
| Molise | 6,289 | 0 | 6,470 | -25 | -25,925 | -7,633 | 16,486 | 14,450 |
| Campania | -6,961 | 9,800 | -8,673 | -2,283 | -19,765 | -5,577 | 10,725 | 9,401 |
| Puglia | -1,477 | 2,205 | -0,228 | -4,625 | -15,151 | -3,855 | 6,777 | 5,940 |
| Basilicata | 7,089 | -0,374 | 4,642 | 2,777 | -31,506 | -3,474 | 15,906 | 13,941 |
| Calabria | 38,262 | 10,691 | -2,801 | -5,454 | -16,402 | -10,446 | 18,298 | 16,039 |
| Sicilia | 0,339 | -3,152 | -18,587 | 19,279 | -6,803 | -1,785 | 13,762 | 12,062 |
| Sardegna | 2,531 | 6,839 | -7,614 | 7,511 | -14,824 | -1,111 | 9,762 | 8,556 |

**C**
