# Supplementary material for: Nationwide analysis of open groin hernia repairs in Italy from 2015 to 2020
Source: Hernia. 2023 Oct 17;27(6):1429–37. doi: 10.1007/s10029-023-02902-z (PMC10700422; doi:10.1007/s10029-023-02902-z)
Supplement: Supplementary file 4 — Supplementary file4 (DOCX 944 KB) [file 10029_2023_2902_MOESM4_ESM.docx]

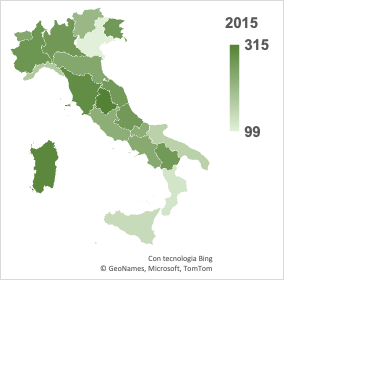

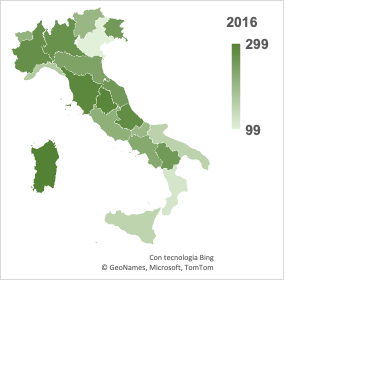


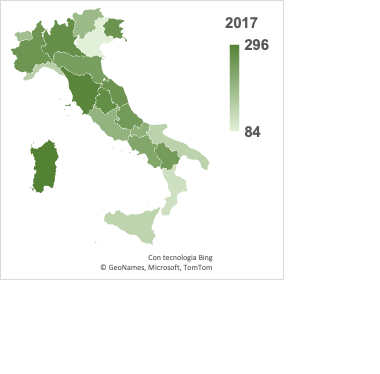

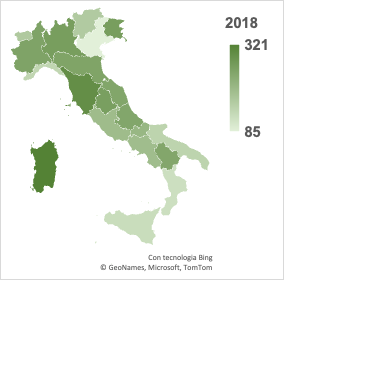


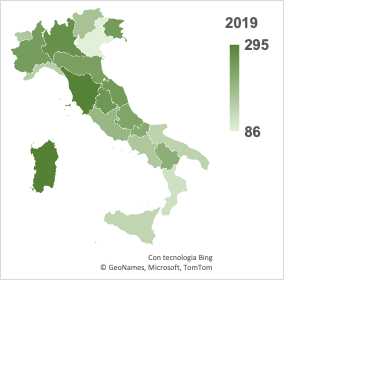

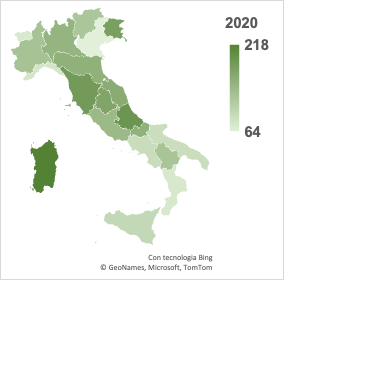


***Supplemental Figure 1*** Annual intervention rate (AIR) of elective open groin hernia procedures (100,000 inhabitants) in Italian population from 2015 to 2020 (sources Agenas and Italian National Institute of Statistics (2019) Resident population on 31st December. ISTAT. <http://dati.istat> .it/?lang=en#.) The AIR was calculated on the total number of unilateral and bilateral procedures performed within the Italian regions and macroregions. The overall mean AIR for elective repairs ranged from 20 to 60 procedures per 100,000 population, with a minimum and maximum of 64 and 321 procedures per 100,000 population in 2020 and 2018 respectively
