# Supplementary material for: Nationwide analysis of open groin hernia repairs in Italy from 2015 to 2020
Source: Hernia. 2023 Oct 17;27(6):1429–37. doi: 10.1007/s10029-023-02902-z (PMC10700422; doi:10.1007/s10029-023-02902-z)
Supplement: Supplementary file 5 — Supplementary file5 (DOCX 630 KB) [file 10029_2023_2902_MOESM5_ESM.docx]

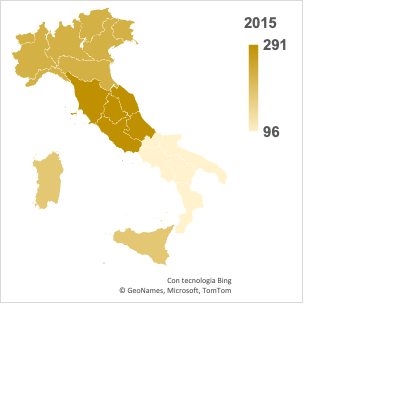

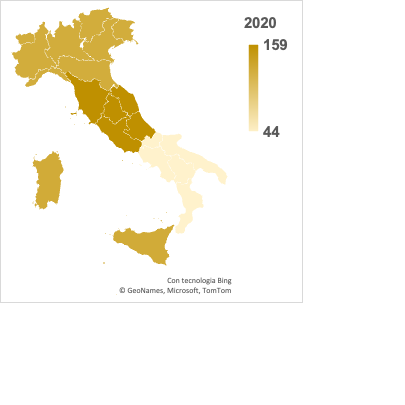
 A


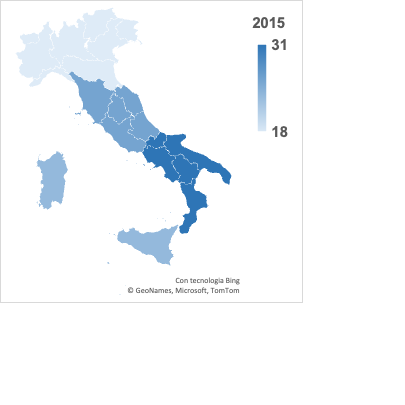

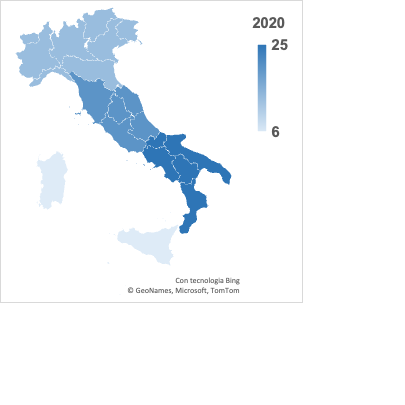
 B

***Supplemental Figure 2*** AIR of elective (A) and urgent (B) open groin hernia procedures (100,000 inhabitants) in Italian population divided in microregion in 2015 and 2020 (sources Agenas and Italian National Institute of Statistics (2019) Resident population on 31st December. ISTAT. <http://dati.istat> .it/?lang=en#.)

Considering the macroregions, the mean AIR ranged from 80 in Southern Italy to 236 procedures in Central Italy per 100,000 population, with a minimum of 44, registered in Southern Italy in 2020, and a maximum of 291 procedures, in Central Italy in 2015, per 100,000 population (Supplemental ***Figure 2***; Supplemental table 2)
