# Supplementary material for: Nationwide analysis of open groin hernia repairs in Italy from 2015 to 2020
Source: Hernia. 2023 Oct 17;27(6):1429–37. doi: 10.1007/s10029-023-02902-z (PMC10700422; doi:10.1007/s10029-023-02902-z)
Supplement: Supplementary file 6 — Supplementary file6 (DOCX 942 KB) [file 10029_2023_2902_MOESM6_ESM.docx]

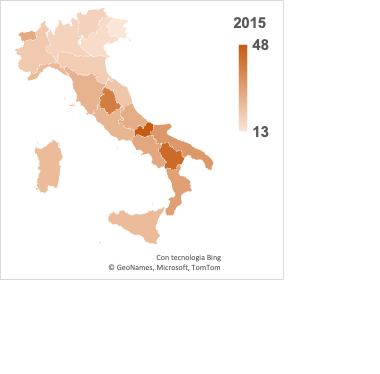

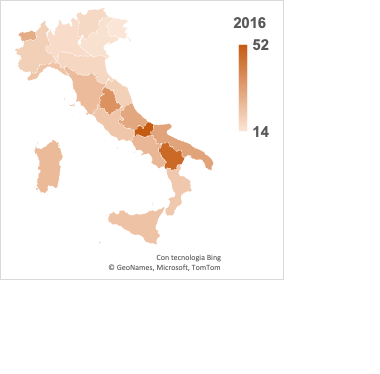


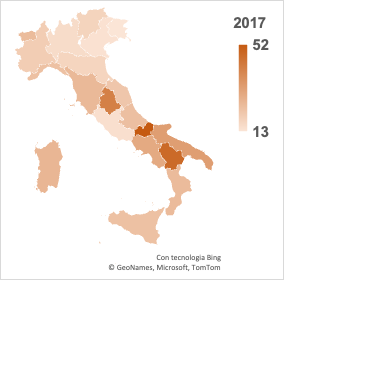

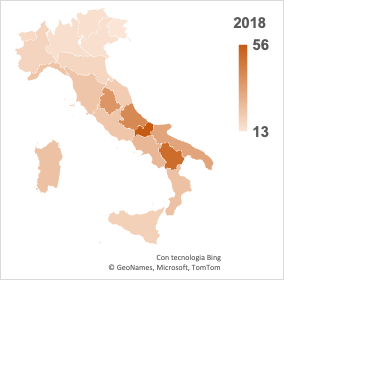


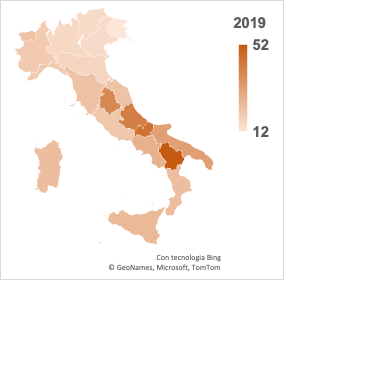

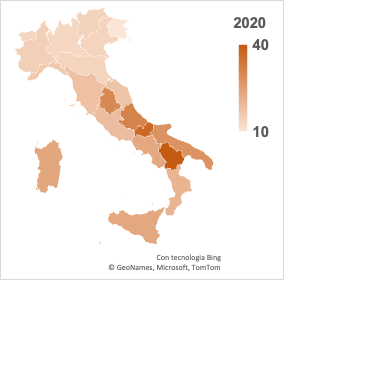


***Supplemental Figure 3***  Annual Interventions Rate (AIR) for urgent open groin hernia procedures (100,000 inhabitants) in Italy from 2015 to 2020 (sources Agenas and Italian National Institute of Statistics (2022) Resident population on 31st December. ISTAT. <http://dati.istat> .it/?lang=en#.) Regarding urgent repairs, the mean AIR ranged from 12 to 48 procedures per 100,000 population, with a minimum and maximum of 10 and 56 procedures per 100,000 population observed in 2020 and 2018 respectively
