# Supplementary material for: Nationwide analysis of open groin hernia repairs in Italy from 2015 to 2020
Source: Hernia. 2023 Oct 17;27(6):1429–37. doi: 10.1007/s10029-023-02902-z (PMC10700422; doi:10.1007/s10029-023-02902-z)
Supplement: Supplementary file 7 — Supplementary file7 (DOCX 941 KB) [file 10029_2023_2902_MOESM7_ESM.docx]

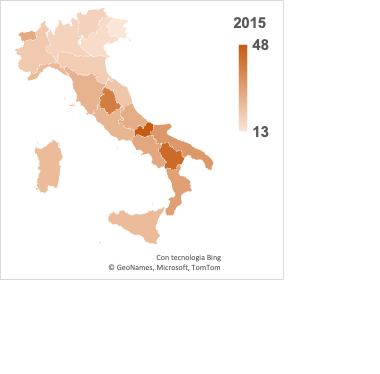

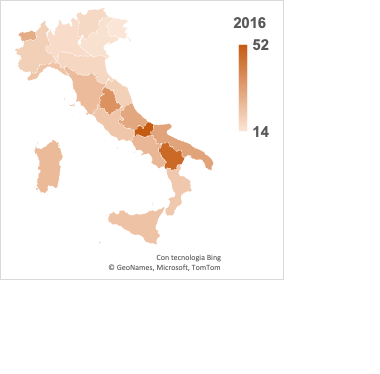


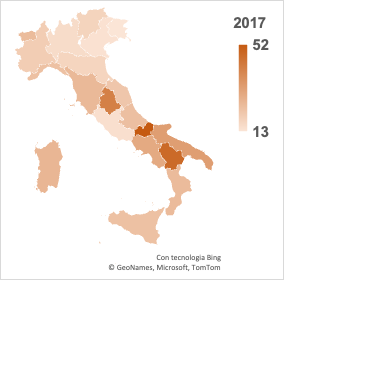

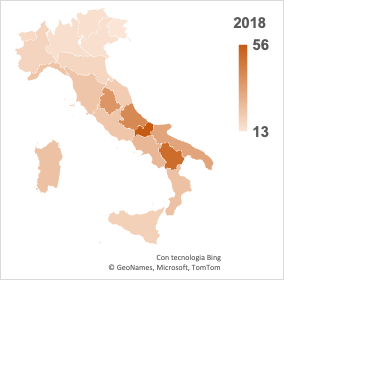


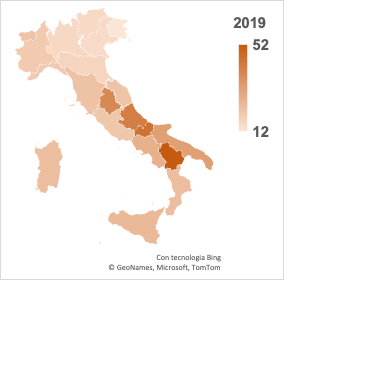

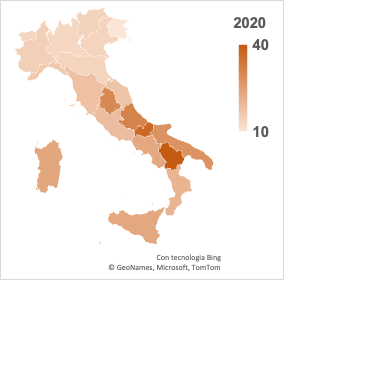


***Figure 11*** Annual Interventions Rate (AIR) for urgent open groin hernia procedures (100,000 inhabitants) in Italy from 2015 to 2020 (sources Agenas and Italian National Institute of Statistics (2022) Resident population on 31st December. ISTAT. <http://dati.istat> .it/?lang=en#.)(Supplemental Referring to macroregions, the mean AIR ranged from 16 in Northern Italy to 29 in Central Italy (minimum=6, registered in the Islands regions in 2020; maximum=31, in Southern Italy observed in 2015 and 2017) procedures per 100,000 population
