# Supplementary material for: Nationwide analysis of open groin hernia repairs in Italy from 2015 to 2020
Source: Hernia. 2023 Oct 17;27(6):1429–37. doi: 10.1007/s10029-023-02902-z (PMC10700422; doi:10.1007/s10029-023-02902-z)
Supplement: Supplementary file 8 — Supplementary file8 (DOCX 19 KB) [file 10029_2023_2902_MOESM8_ESM.docx]

Supplemental Table 4 Annual trend for elective (A) and urgent (B) procedures per 100,000 inhabitants

|  | ANNUAL TREND | | | | | | |
| --- | --- | --- | --- | --- | --- | --- | --- |
| REGION | **2016** | **2017** | **2018** | **2019** | **2020** | ***p*** | ***p^1^*** |
| PPiemonte | -1.119 | -6,624 | -4,193 | -2,258 | -93,218 | <0.0001 | <0.0001 |
| Valle d'Aosta | -16,165 | -16,157 | -8,019 | -4,950 | -119,565 | 0.788 | 0.860 |
| Lombardia | -0,749 | 0,175 | -2,664 | 2,341 | -82,529 | <0.0001 | 0.493 |
| PA di Bolzano | -2,004 | -12,057 | -8,462 | -11,908 | -53,524 | 0.102 | 0.122 |
| PA di Trento | -3,560 | -3,691 | 1,688 | -1,979 | -32,952 | 0.530 | 0.757 |
| Veneto | -0,580 | -18,115 | 1,566 | 1,120 | -33,662 | 0.0003 | 0.034 |
| Friuli Venezia Giulia | -6,328 | 1,550 | 0,063 | -5,643 | -41,541 | 0.047 | 0.9243 |
| Liguria | -4,516 | -15,313 | -5,248 | 6,391 | -90,555 | 0.048 | 0.034 |
| Emilia-Romagna | -1,887 | 1,175 | 3,213 | -3,677 | -57,617 | <0.0001 | <0.0001 |
| Toscana | -1,983 | -0,187 | 1,516 | -0,350 | -59,170 | 0.942 | 0.069 |
| Umbria | -8,107 | -7,283 | -5,952 | -1,887 | -52,257 | <0.0001 | <0.0001 |
| Marche | -4,790 | 0,527 | -7,291 | 1,589 | -58,232 | 0.001 | 0.010 |
| Lazio | -6,006 | -7,588 | -1,790 | -1,295 | -39,358 | <0.0001 | 0.124 |
| Abruzzo | 2,861 | -11,993 | -3,441 | -6,775 | -21,151 | 0.704 | 0.109 |
| Molise | -15,097 | 2,377 | -0,159 | 3,817 | -47,191 | 0.164 | 0.153 |
| Campania | -4,046 | -1,059 | -17,283 | -19,362 | -80,710 | 0.001 | 0.001 |
| Puglia | -4,821 | -9,576 | 2,515 | -4,631 | -55,827 | 0.119 | 0.060 |
| Basilicata | -5,681 | -4,208 | -3,469 | -15,911 | -69,913 | 0.116 | 0.002 |
| Calabria | -3,592 | -4,342 | 5,608 | -7,414 | -52,294 | 0.255 | 0.395 |
| Sicilia | 6,082 | -9,927 | -9,823 | 6,743 | -41,170 | <0.0001 | 0.027 |
| Sardegna | -1,633 | -1,282 | 7,036 | -9,424 | -37,439 | <0.0001 | <0.0001 |

**A**

|  | ANNUAL TREND | | | | |  |  |
| --- | --- | --- | --- | --- | --- | --- | --- |
| REGION | **2016** | **2017** | **2018** | **2019** | **2020** | ***p*** | ***p^1^*** |
| Piemonte | 1,353 | -2,900 | -8,428 | 5,470 | -35,427 | 0.022 | 0.223 |
| Valle d'Aosta | 5,405 | -19,355 | -47,619 | 22,222 | -68,750 | 0.926 | 0.691 |
| Lombardia | -4,423 | -6,290 | -2,208 | 1,535 | -23,930 | *0.005* | 0.097 |
| PA di Bolzano | -14,286 | 14,953 | -24,419 | 0,000 | -8,861 | 0.666 | 0.342 |
| PA di Trento | 14,737 | -11,765 | -14,865 | 1,333 | -10,294 |  |  |
| Veneto | 2,906 | -10,029 | 6,395 | -1,801 | -11,077 | 0.339 | 0.640 |
| Friuli Venezia Giulia | 10,920 | -9,434 | 0,000 | -12,766 | -21,552 | 0.008 | 0.001 |
| Liguria | -1,657 | -1,117 | 0,556 | -20,805 | -22,131 | 0.597 | 0.442 |
| Emilia-Romagna | -4,120 | -2,430 | 2,005 | -8,571 | -22,705 | 0.080 | 0.204 |
| Toscana | -0,841 | -1,386 | -10,613 | -5,604 | -23,349 | 0.011 | 0.0001 |
| Umbria | -7,764 | 11,295 | -10,000 | 1,493 | -29,344 | 0.815 | 0.522 |
| Marche | -2,597 | 6,667 | -4,430 | 1,250 | -27,490 | 0.001 | 0.008 |
| Lazio | -9,404 | -54,610 | 35,222 | -10,772 | -10,808 | 0.095 | 0.018 |
| Abruzzo | 12,099 | -31,494 | 42,963 | -0,372 | -35,176 | 0.925 | 0.633 |
| Molise | 6,289 | 0,000 | 6,471 | -25,000 | -25,926 | 0.826 | 0.715 |
| Campania | -6,962 | 9,800 | -8,673 | -2,283 | -19,766 | 0.001 | 0.061 |
| Puglia | -1,477 | 2,205 | -0,229 | -4,625 | -15,152 | <0.0001 | <0.0001 |
| Basilicata | 7,090 | -0,375 | 4,643 | 2,778 | -31,507 | 0.958 | 0.622 |
| Calabria | -38,263 | 10,692 | -2,802 | -5,455 | -16,402 | 0.591 | 0.437 |
| Sicilia | 0,340 | -3,152 | -18,588 | 19,279 | -6,804 | 0.060 | 0.051 |
| Sardegna | 2,532 | 6,840 | -7,614 | 7,512 | -14,825 | 0.039 | 0.073 |

**B**
